# Synthesis of BSA-Coated Iron Oxide Nanoparticles with Size Control for High-Performance *T*_1_ Contrast Agents in Magnetic Resonance Imaging

**DOI:** 10.3390/biom16030478

**Published:** 2026-03-23

**Authors:** Bosede Kolawole, Jie Zheng, Dongmei Cao, Yongfeng Zhao

**Affiliations:** 1 Department of Chemistry, Physics and Atmospheric Science, Jackson State University, Jackson, MS 39217, USA; bosede.o.kolawole@students.jsums.edu; 2 Mallinckrodt Institute of Radiology, Washington University School of Medicine, St. Louis, MO 63110, USA; zhengj@wustl.edu; 3 Advanced Microscopy and Analytical Core, Louisiana State University, Baton Rouge, LA 70803, USA; dcao2@lsu.edu

**Keywords:** chemical co-precipitation, bovine serum albumin, *T*_1_ contrast agents

## Abstract

The excellent biocompatibility and favorable physicochemical properties of iron oxide nanoparticles have made them attractive candidates for magnetic resonance imaging. However, it remains challenging to synthesize high-performance *T*_1_ contrast agents with controlled sizes and biocompatible coating materials. In this study, we demonstrate a simple and environmentally friendly approach for synthesizing ultra-small iron oxide nanoparticles using bovine serum albumin (BSA) as a template. Following synthesis, the iron oxide nanoparticles (Fe_3_O_4_) were oxidized to Fe_2_O_3_ via the addition of hydrogen peroxide, which resulted in enhanced *T*_1_-weighted magnetic resonance contrast. The use of BSA not only stabilized the nanoparticles but also enabled precise control over nanoparticle size by adjusting the Fe-to-BSA molar ratio. This method yielded highly uniform and crystalline ultra-small nanoparticles ranging from approximately 3.7 to 7.9 nm in diameter. The *T*_1_ contrast performance of the Fe_2_O_3_@BSA nanoparticles was evaluated at 3 T magnetic field. Among the synthesized samples, nanoparticles with sizes of 4.6 nm exhibited the strongest *T*_1_ contrast enhancement along with low *r*_2_/*r*_1_ ratios. These features highlight their potential as promising alternatives to gadolinium-based contrast agents. In addition to their superior performance, this synthesis method is low-cost and non-toxic, making it suitable for scalable biomedical applications.

## 1. Introduction

Magnetic resonance imaging (MRI) is a widely used diagnostic tool in clinical practice [1]. Besides its non-invasive nature, MRI can provide high-resolution images of internal body structures without the use of ionizing radiation [1,2,3,4,5]. By employing strong magnetic fields and radiofrequency pulses, MRI generates detailed two- and three-dimensional tomographic images based on the nuclear magnetization of hydrogen protons in tissues. Its excellent soft-tissue contrast and high spatial resolution make MRI a standard for imaging of the brain, spine, cardiovascular system, musculoskeletal tissues, and oncology [6]. Clinical MRI contrast primarily relies on intrinsic *T*_1_ and *T*_2_ relaxation properties of tissues, which are often amplified by contrast agents to enhance diagnostic sensitivity.

To further improve image contrast, MRI contrast agents are routinely used and are classified as either positive (*T*_1_-weighted) or negative (*T*_2_- weighted) agents based on their influence on signal intensity [7]. Positive contrast agents are typically gadolinium-based chelates or manganese complexes, whereas superparamagnetic iron oxide nanoparticles have traditionally been employed as negative contrast agents due to their strong *T*_2_-shortening effects [3,7,8]. Contrast agents enhance image quality by accelerating proton nuclear spin relaxation, thereby improving signal intensity and lesion detection [9].

Gadolinium-based contrast agents (GBCAs) have been extensively used in clinical MRI [10]. However, growing safety concerns have limited their long-term application. Adverse effects associated with GBCAs include nephrogenic systemic fibrosis (NSF) in patients with impaired renal function and long-term gadolinium retention in the brain and other tissues [11]. Additionally, GBCAs are rapidly cleared through renal excretion, which restricts their use in prolonged or repeated imaging. These limitations have motivated intensive research for safer alternatives to gadolinium-based contrast agents [12,13,14].

Iron oxide nanoparticles offer several advantages as MRI contrast agents, including metabolism through natural iron pathways, high relaxivity, and improved biocompatibility [15,16]. Certain iron oxide formulations have demonstrated superior imaging performance compared to commercial gadolinium chelates, even at low and intermediate magnetic field strengths. Clinically, iron oxide nanoparticles have been successfully used for liver and lymph node imaging, owing to their uptake by the reticuloendothelial system and incorporation into ferritin and normal iron metabolism [16,17]. Due to their favorable safety profile and high relaxivity, iron oxide nanoparticles are increasingly being explored as potential alternatives to GBCAs, particularly as *T*_1_ contrast agents [4].

The magnetic behavior and relaxivity of iron oxide nanoparticles are strongly dependent on their size, crystallinity, magnetic anisotropy, hydrodynamic diameter, and surface coating [18,19]. These parameters collectively regulate water accessibility and local magnetic field fluctuations [20]. Conventional iron oxide nanoparticles with core sizes of approximately 8–30 nm generate strong local magnetic field inhomogeneities. As a result, transverse relaxation is predominantly accelerated, leading to enhancement on *T*_2_-weighted images [21,22]. Clinically approved agents such as ferumoxides, ferucarbotran, and ferumoxtran exhibit high *r*_2_/*r*_1_ ratios, making them effective *T*_2_ contrast agents for liver and lymph node imaging [21]. At standard clinical field strengths (1.5–3 T), the high magnetic susceptibility of these particles favors transverse over longitudinal relaxation, limiting their utility as *T*_1_ enhancers [16].

Recent studies have demonstrated that ultra-small iron oxide nanoparticles with core sizes below ~5 nm can exhibit strong *T*_1_ contrast due to their reduced magnetic moments and enhanced longitudinal relaxation [23,24]. Nanoparticles with low *r*_2_/*r*_1_ ratios and high *r*_1_ values typically possess moderate magnetization and thin water-permeable surface coatings that promote efficient proton exchange near the magnetic core [20,23]. Exceedingly small iron oxide nanoparticles with hydrodynamic diameters below the renal clearance threshold (~5.5 nm) have shown *r*_2_/*r*_1_ ratios approaching those of GBCAs, with comparable *r*_1_ values and improved clearance profiles [23].

The synthesis method plays a critical role in determining the size, crystallinity, morphology, and magnetic properties of iron oxide nanoparticles [25,26,27]. While thermal decomposition and hydrothermal methods can produce well-defined nanoparticles, they often require organic solvents, high temperatures, or high pressures [28]. In contrast, the co-precipitation method is a green and scalable approach that utilizes aqueous media at relatively low temperatures. Hydrophilic nanoparticles are produced by precipitating Fe^2+^ and Fe^3+^ salts under basic conditions [29]. However, co-precipitation typically suffers from limited control over particle size and size uniformity.

To address this limitation, various surface-modifying agents have been explored to regulate nanoparticle growth during co-precipitation. Natural fatty acids, synthetic polymers such as poly(acrylic acid), and proteins including bovine serum albumin (BSA) have been shown to influence nanoparticle size and morphology [30,31,32]. Among these, BSA is particularly attractive due to its biocompatibility, non-toxicity, and widespread clinical use. BSA coatings enhance colloidal stability, prolong circulation time, and provide functional groups for surface modification [21,33]. In MRI and magnetic particle imaging, albumin-coated iron oxide nanoparticles have demonstrated improved stability under physiological conditions while maintaining favorable magnetic properties.

Although BSA has previously been used to synthesize ultra-small iron oxide nanoparticles [31], systematic control of iron oxide core size and its direct impact on *T*_1_ contrast performance have not been explored [31,34,35]. In particular, the interplay between BSA concentration, core size, and resulting *r*_1_ and *r*_2_/*r*_1_ values has not been fully elucidated.

In this study, we employ BSA as a size-regulating agent to synthesize iron oxide nanoparticles via the co-precipitation method by systematically varying the Fe:BSA molar ratio. We investigate how controlled changes in nanoparticle size influence *T*_1_ relaxivity and overall MRI performance, aiming to optimize iron oxide nanoparticles as effective and safe *T*_1_ contrast agents.

## 2. Materials and Methods

### 2.1. Materials and Reagents

Bovine serum albumin (BSA), FeCl_3_∙6H_2_O, FeSO_4_∙7H_2_O, NaOH, and hydrogen peroxide (35%) were puchased from Sigma-Aldrich (St. Louis, MO, USA). All chemicals were used without purification.

### 2.2. Synthesis of Fe_3_O_4_@BSA and Fe_2_O_3_@BSA

In a typical reaction, BSA (30.23 mg, 0.00045 mmol) in 10 mL of deionized water was purged with argon for 30 min. FeCl_3_∙6H_2_O (27.03 mg, 0.1 mmol) and FeSO_4_∙7H_2_O (13.9 mg, 0.05 mmol) were dissolved in 2 mL of deionized water and were injected into the BSA solution and purged for another 15 min. The reaction mixture was placed in a water bath (60 °C) and allowed to heat up for 5 min. NaOH solution (0.5 ml, 0.6 m mol) was added at 12 mL/hr, and allowed to stir for an hour. The Fe_3_O_4_@BSA (6 ml) was taken out and H_2_O_2_ peroxide (35%) (0.5 ml) was added at 12 mL/hr under low stirring for the formation of Fe_2_O_3_@BSA. The reaction was stirred for 10–30 min and allowed to settle down overnight. To purify, the reaction mixture was washed with deionized water amicon filter (100 k) until the pH reached 7. The same reaction was repeated with different amounts of BSA (60.45 mg, 120.90 mg, and 237.7 mg) for 165:1, 83:1, 43:1 Fe:BSA molar ratio respectively.

### 2.3. Characterization of Fe_3_O_4_@BSA and Fe_2_O_3_@BSA

#### 2.3.1. Transmission Electron Microscope

The information about the sizes and morphology of the nanoparticles was obtained using a transmission electron microscope (TEM) (JEOL JEM-1011, JEOL, Inc., Peabody, MA, USA). The diluted purified samples (4 µL) were placed on a Carbon film 150-mesh copper TEM grid (CF 150-CU) and allowed to stay for 20 min. Excess samples were removed using a small piece of filter paper and allowed to dry completely before viewing them under the transmission electron microscope. About 100 nanoparticles were analyzed using ImageJ (v1.54d, National Institutes of Health, Bethesda, MD, USA) software to analyze the TEM images. The statistical analyses were conducted in Origin 2008.

#### 2.3.2. Crystal Size Measurement of Iron Oxide Nanoparticles

X-ray diffraction (XRD) measurement was done using the Rigaku MiniFlex 600 X-ray (40 kV, 15 mA) (Rigaku Americas Corp, The Woodlands, TX, USA) with Cu K*β* radiation (λ = 0.154 nm). The samples were placed on zero-background quartz slides and allowed to dry. Then, the samples were mounted on the central goniometer stage (beam incident position) of the XRD instrument. The scan degree ranged from 10° to 80° with a step size of 0.01 at a rate of 1°/min. The crystal sizes of the nanoparticles were calculated using the Scherrer equation.

#### 2.3.3. X-Ray Photoelectron Spectroscopy (XPS)

X-ray Photoelectron Spectroscopy (XPS) analysis of the samples was conducted using a ScientaOmicron ESCA 2SR X-ray Photoelectron Spectroscope equipped with a flood source charge neutralizer. The powders were pressed into pellets and analyzed using a monochromatic Al Kα X-ray source (1486.6 eV) at 450 W, with the analysis chamber pressure maintained below 5 × 10^−9^ mBar. Both wide-scan surveys and high-resolution core-level spectra of all elements were acquired, with binding energies calibrated using the major N 1s peak of BSA protein at 399.8 eV. The core-level spectra were deconvoluted to obtain chemical state information using CasaXPS (v2.3.25, Casa Software) data processing software.

#### 2.3.4. Hydrodynamic Size

The hydrodynamic diameter and size distribution of the nanoparticles were measured by dynamic light scattering (DLS) using a Litesizer^TM^ 100 Anton Paar particle size analyzer (Anton Paar USA Inc., Ashland, VA, USA). Prior to measurement, samples were dispersed in deionized water and appropriately diluted to minimize multiple scattering effects. All measurements were performed at room temperature and the reported values represent the number-weighted average hydrodynamic diameter.

#### 2.3.5. UV Spectrometer

UV-Vis absorption spectroscopy was used to confirm the presence of bovine serum albumin (BSA) on the surface of the iron oxide nanoparticles. Spectra were recorded using a Shimadzu UV-Vis spectrophotometer (Shimadzu Corporation, Kyoto, Japan). Stock dispersions of BSA-coated iron oxide nanoparticles (2 mM) were diluted into deionized water to a final concentration of 0.4 mM. Aliquots of 2 mL were prepared in quartz cuvettes (1 cm path length). For comparison and confirmation of BSA coating, a reference solution of pure BSA was prepared by dissolving a small amount of BSA in ultra-pure water at equivalent protein concentration (e.g., 0.1–1 mg/mL). Both samples and the BSA reference were gently pipetted to ensure homogeneity and degassed to remove air bubbles or BSA foam. Cuvettes were rinsed 3 times with deionized water before filling. Deionized water served as the blank. Absorbance spectra were recorded from 200 to 800 nm to identify characteristic BSA protein absorption bands (~280 nm) alongside nanoparticle plasmonic features, confirming successful protein coating on the iron oxide cores. The BSA functionalized nanoparticles exhibited a characteristic absorption band around 280 nm attributed to the aromatic amino acid residues (trptophan and tyrosine) of BSA, which was absent in the spectra of bare iron oxide nanoparticles.

#### 2.3.6. Fourier Transform Infrared Spectroscopy (FTIR)

A Perkin Elmer Fourier Transform Infrared (FTIR) Spectrometer (Perkin Elmer, Waltham, MA, USA) was used to scan the dried nanoparticles. Drops (~5–10 µL) of concentrated BSA-coated iron oxide nanoparticle dispersion, pure BSA solution, and uncoated iron oxide nanoparticle dispersion were separately deposited on clean KBr crystals and allowed to air-dry completely at room temperature to form thin films. Dried samples were then mounted in the FTIR instrument for measurement. Spectra were recorded in the 400–4000 cm^−1^ range at 4 cm^−1^ resolution with 64 scans averaged. FTIR was also used to confirm BSA functional groups.

#### 2.3.7. Determination of Iron Concentration

The iron concentration of the nanoparticles was determined using an Agilent 5800 VDV inductively coupled plasma optical emission spectrometer (ICP-OES; Agilent Technology, Santa Clara, CA, USA) equipped with an SPS 4 autosampler and a recirculating chiller. The instrument was operated under manufacturer-recommended conditions. Briefly, an aliquot of the stock dispersion (5 µL, containing approximately 3.5 mg/mL iron concentration) was transferred into a 0.5 mL centrifuge tube containing 95 µL of deionized water. Aqua regia (HCl and HNO3, 3:1) (100 µL) was added and sonicated for 30 min for complete digestion of the iron oxide core and BSA coating of the sample. A freshly prepared dilution matrix (~0.5% HCl, 5% HNO_3_) was used to dilute the samples to the range of 0.5–0.8 ppm. Calibration standards in the range of 0.125, 0.25, 0.5, 1, and 2 ppm Fe were prepared from a certified 200 ppm Fe standard using the same acid matrix solution (~0.5% HCl, 5% HNO_3_). All standards were prepared fresh daily, measured in triplicate alongside digested nanoparticle samples to construct a linear calibration curve (R^2^ > 0.999). Sample iron concentrations were determined by interpolation from this calibration curve and corrected for all serial dilution factors applied during sample preparation. Data acquisition and quantitative analysis were performed using ICP ExpertTM software (v7.6.2, Agilent Technologies). Quality control checks were conducted to ensure measurement accuracy and reproducibility.

#### 2.3.8. In Vitro MRI Relaxivity Study

Relaxivity measurements were performed on a 3T clinical Prisma MRI scanner (Siemens Heathineers, Erlangen, Germany). Aliquots (2 mL) of BSA-coated iron oxide nanoparticle dispersions at iron concentrations of 0.8, 0.4, 0.2, 0.1, and 0.05 mM were prepared in 4 mL glass vials and arranged in a multi-sample phantom holder. Vials were positioned at the scanner isocenter using a dedicated head coil with room temperature at 22 °C. *T*_1_ and *T*_2_ relaxation times were determined using standard sequences. A series of *T*_1_-weighted images was acquired by a 2D inversion-recovery spin-echo pulse sequence at multiple times of inversion (TIs) (TR = 7000 ms, TI = 50, 150, 300, 450, …, 2500 ms, slice thickness = 5 mm). *T*_2_-weighted images were acquired by a 2D multi-echo *T*_2_-weighted spin-echo pulse sequence (TR = 4000 ms, 10 TEs from 10 ms to 100 ms at a step of 10 msec, slice thickness = 5 mm). Imaging field of view (FOV) was 180 × 90 mm and the matrix size was 192 × 96. The specific relaxivities of *r*_1_ and *r*_2_ (mM^−1^s^−1^) for nanoparticles were computed by taking the linear slope of 1/*T*_2_ (or 1/*T*_1_) versus Fe concentration.

## 3. Results and Discussion

### 3.1. Synthesis and Characterization

**Figure 1:**
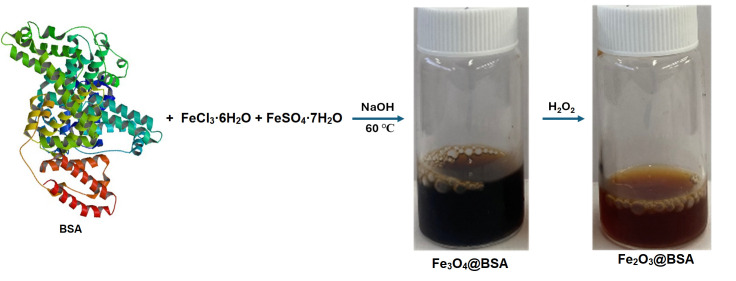
Schematic illustration of the synthesis of Fe_3_O_4_@BSA and Fe_2_O_3_@BSA nanoparticles.

The Fe_3_O_4_@BSA nanoparticles were synthesized through the co-precipitation method as outlined in Figure 1. Co-precipitation is a commonly used approach for fabrication of Fe_3_O_4_ nanoparticles because the reaction is carried out in aqueous solutions under relatively low temperatures. No further surface modification is required to achieve water dispersibility. Due to its high compatibility and common availability, BSA was used as template and surface coating material in the mixture of ferric and ferrous salt.

**Figure 2:**
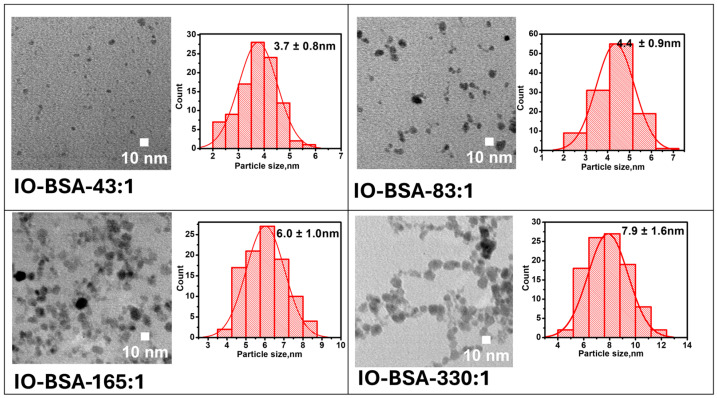
Transmission electron microscopy (TEM) images of Fe_3_O_4_@BSA with their size distribution.

In the synthesis, BSA solution was mixed with ferric and ferrous salt solution (molar ratio of 2:1). After purging with argon gas, NaOH solution was added slowly under stirring at an elevated temperature. To control the size of iron oxide nanoparticles, the relative ratio of iron and BSA was adjusted. The size was characterized by TEM after purification (Figure 2). The effect of BSA in the control of the nanoparticle size is clearly observed in the TEM images. The sizes obtained at different Fe:BSA molar ratios of 43:1, 83:1, 165:1, and 330:1 are 3.7 ± 0.1 nm, 4.4 ± 0.9 nm, 6.0 ± 1.0 nm, and 7.9 ± 1. 5 nm respectively. The particle sizes increase with decreasing BSA. Interestingly, when NH_4_OH was used instead of NaOH, the nanoparticle size could not be tuned by varying the Fe:BSA ratio. 

**Figure 3:**
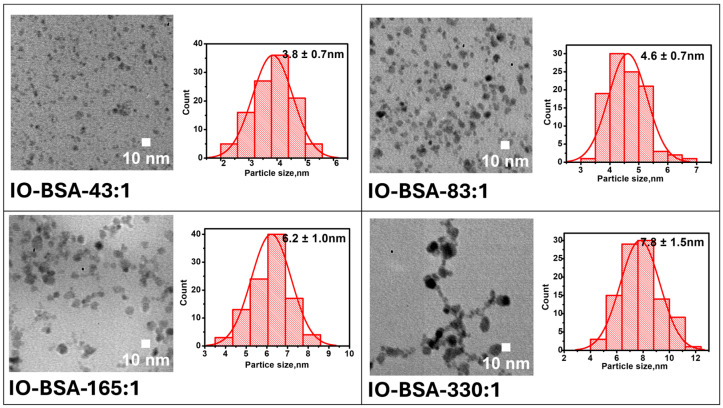
TEM images of Fe_2_O_3_@BSA with size distribution.

Studies have reported that Fe_2_O_3_ nanoparticles are better *T*_1_ contrast agents than Fe_3_O_4_ [17]. To study the *T*_1_ imaging property, the resulting Fe_3_O_4_ was further oxidized with hydrogen peroxide. Upon the oxidation of Fe_3_O_4_@BSA to Fe_2_O_3_@BSA, there was no significant change in the sizes of the iron oxide nanoparticles (Figure 3). The sizes at different Fe:BSA molar ratios—43:1, 83:1, 165:1, and 330:1—were 3.8 ± 0.7 nm, 4.6 ± 0.7 nm, 6.2 ± 0.9 nm, and 7.8 ± 1. 5 nm respectively.

### 3.2. XRD and XPS

**Figure 4:**
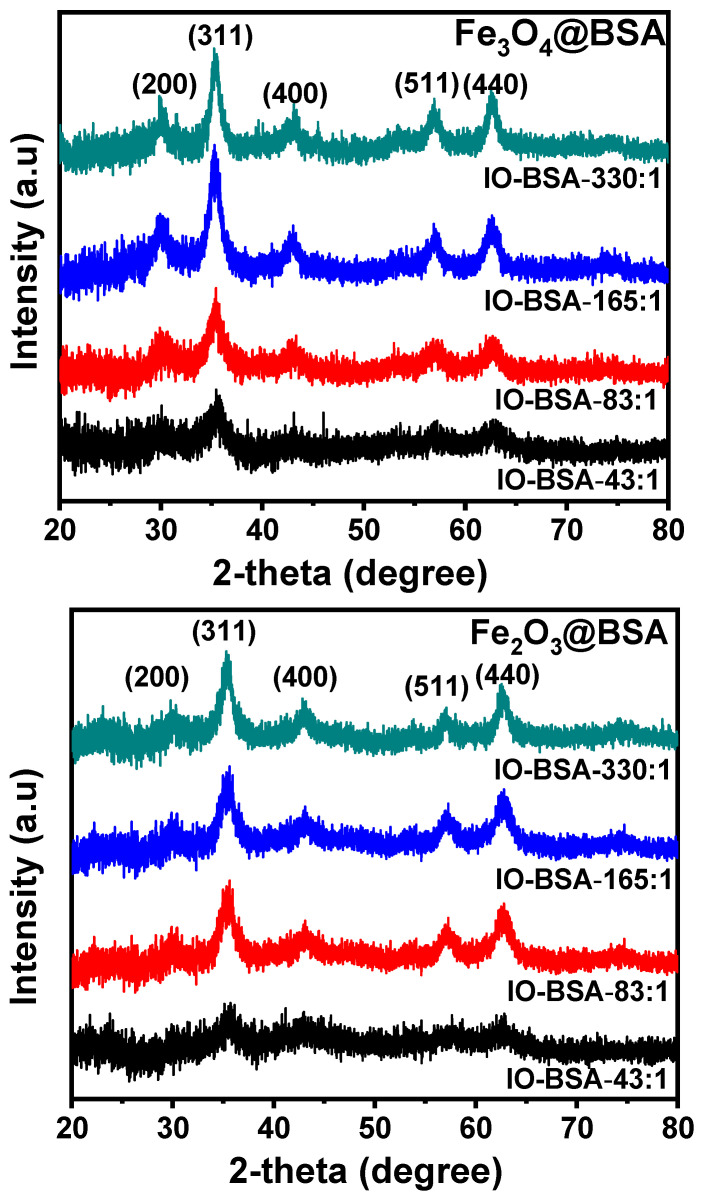
Powder X-ray diffraction (XRD) patterns of Fe_3_O_4_@BSA and Fe_2_O_3_@BSA with different Fe:BSA molar ratios.

The crystal structure of the nanoparticles was further studied by X-ray diffraction (XRD). From the XRD data (Figure 4), the characteristic diffraction peaks for the nanoparticles are clearly observed, and these diffraction peaks align well with the theoretical patterns (JCPDS 19-0629). Indicated by the relatively sharp diffraction peaks, the nanoparticles appear to be highly crystalline. In addition, the peak intensity increases with increasing particle size. According to the Scherrer equation, the crystal sizes of the nanoparticles for Fe_3_O_4_@BSA with different Fe:BSA molar ratios—43:1, 83:1, 165:1, and 330:1—are calculated as 3.9, 4.9, 6.6, and 7.0 nm respectively, while those of Fe_2_O_3_@BSA are 4.0, 5.6, 6.9, and 7.9 nm respectively. The crystal sizes are close to the values from the TEM images, confirming the size control of iron oxide nanoparticles using BSA. Based on XRD data, Rietveld refinement and the Le Bail method were reported to be employed to extract structural parameters [36]. However, the lattice parameters of Fe_3_O_4_ and Fe_2_O_3_ are very similar and are widely reported to be difficult to distinguish [36,37]. In addition, the nanoparticles synthesized in this study are ultra-small and coated with BSA. The XRD patterns exhibit significant peak broadening, which may reduce the accuracy and reliability of structural parameters obtained from Rietveld refinement.

**Figure 5:**
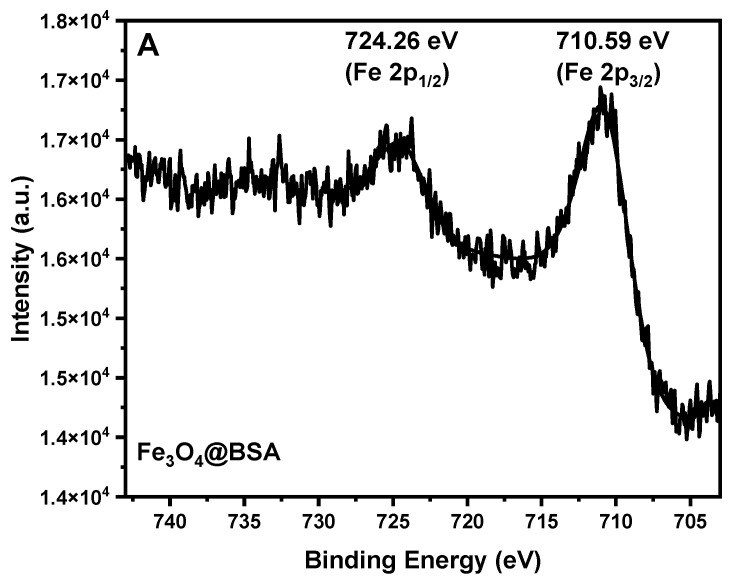

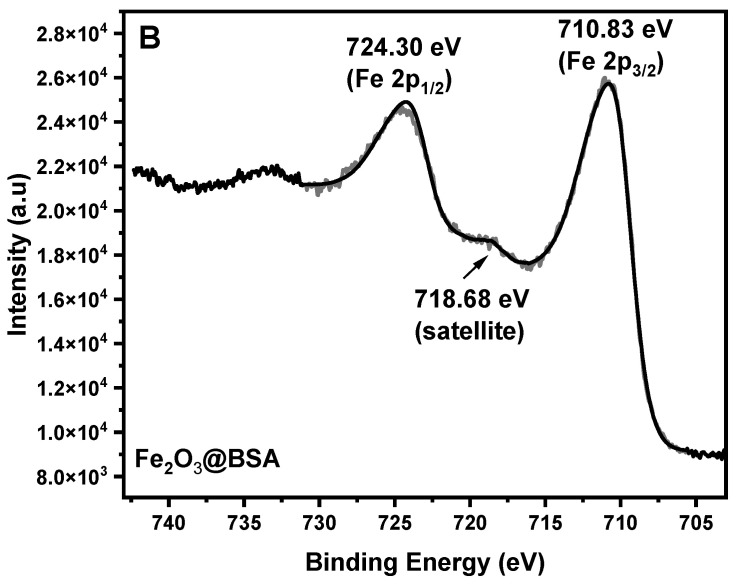
The XPS spectra of Fe 2p for (**A**) Fe_3_O_4_@BSA and (**B**) Fe_2_O_3_@BSA.

To further investigate the different phases, XPS was conducted to characterize the Fe_3_O_4_@BSA and Fe_2_O_3_@BSA nanoparticles. As shown in Figure 5A, two peaks of 710.59 eV (2p_3/2_) and 724.26 eV (2p_1/2_) were observed for the Fe_3_O_4_ phase, which are slightly lower than those of the Fe_2_O_3_ phase [31]. In contrast, two peaks of 710.83 eV (2p_3/2_) and 724.30 eV (2p_1/2_) were observed for Fe_2_O_3_@BSA (Figure 5B), which can be attributed to Fe (III). In addition, a significant satellite peak at 718.68 eV was identified, which further confirms the formation of Fe_2_O_3_ [31,38,39].

### 3.3. FT-IR

**Figure 6:**
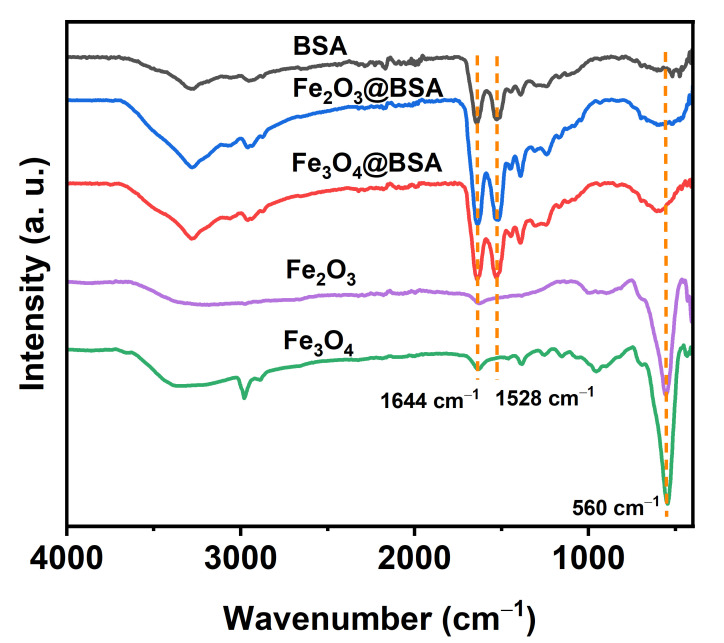
Fourier transform infrared spectroscopy of bovine serum albumin (BSA), Fe_3_O_4_@BSA, Fe_2_O_3_@BSA, Fe_3_O_4_ and Fe_2_O_3_.

Since BSA was used in the synthesis, we expected that BSA was coated on the surface of the nanoparticles. To confirm the coating material on the surface of nanoparticles, Fourier transform infrared (FT-IR) for synthesized nanoparticles was performed. For comparison, the FT-IR for pure BSA was collected as control. As shown in Figure 6, the absorptions of 1644 cm^−1^ and 1528 cm^−1^ correspond to the C=O and N–H stretching vibrations [40]. The broad band at 3000–3600 cm^−1^ corresponds to the O–H and N–H stretching. The absorption at 560 cm^−1^ is attributed to Fe–O vibration. The characteristic peaks of BSA were clearly identified on Fe_2_O_3_@BSA and Fe_3_O_4_@BSA, indicating the surface coating of the iron oxide nanoparticles with BSA.

### 3.4. UV-Vis

**Figure 7:**
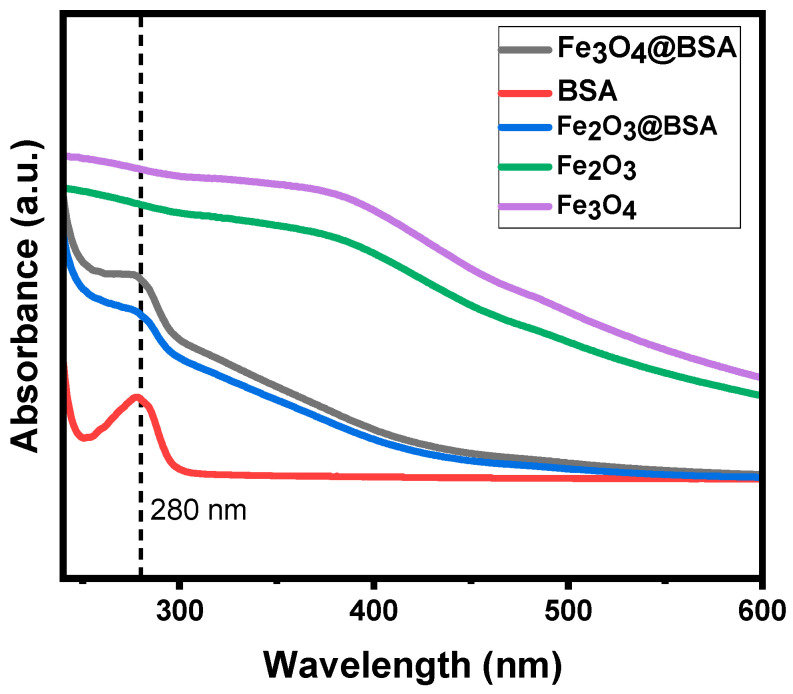
UV spectra of BSA, Fe_3_O_4_@BSA, Fe_2_O_3_@BSA, Fe_3_O_4_ and Fe_2_O_3_ without BSA.

Because BSA typically has UV absorption at 280 nm, the UV spectra of the resulting nanoparticles were collected to confirm their coating materials (Figure 7). Compared with iron oxide nanoparticles without the surface coating, the UV spectra of both Fe_3_O_4_@BSA and Fe_2_O_3_@BSA show the characteristic BSA absorption peak around 280 nm. The UV spectra suggest that the surface coating on the iron oxide nanoparticles is BSA [41]. These results confirm that the nanoparticles consist of iron oxide cores coated with BSA. The exact amount of BSA on each nanoparticle was not concluded in this study, although previous work has reported approximately 6–7 BSA subunits per nanoparticle [31].

### 3.5. Water Dispersibility

**Figure 8:**
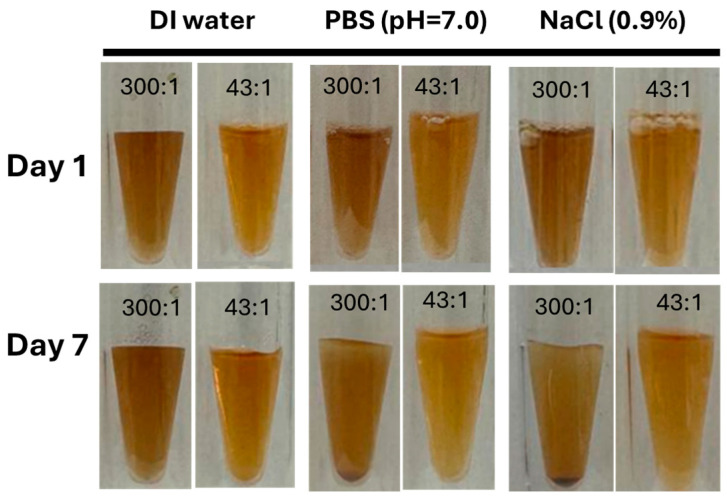
Water stability test of Fe_2_O_3_@BSA with different Fe:BSA (2 mM) ratios of 330:1 and 43:1 (from **left** to **right**) in deionized water, pBS, and NaCl (0.9%).

High water dispersibility plays a key role for the biomedical application of iron oxide nanoparticles. Traditionally, iron oxide nanoparticles with precisely controlled size can be synthesized by thermal decomposition. However, the problem is that the synthesized nanoparticles can only be dispersed in organic solvents. A surface modification is needed to provide water dispersibility [14]. The water dispersibility largely depends on the presence of hydrophilic coating ligands. Bovine serum albumin (BSA) is a water-soluble natural protein and highly biocompatible [42]. BSA contains hydrophilic groups that coordinate with the Fe ions [32]. The coating of BSA on the surface of nanoparticles could significantly improve water dispersibility. As shown in Figure 8, Fe_2_O_3_@BSA nanoparticles of various sizes exhibit excellent dispersibility. Furthermore, the solution is stable up to 7 days in different aqueous media, including DI water, PBS, and NaCl (0.9%), which has a high ion strength. The BSA-coated nanoparticles are highly stable in physiological solution, which is important for biomedical applications.

### 3.6. Hydrodynamic Diameter

**Figure 9:**
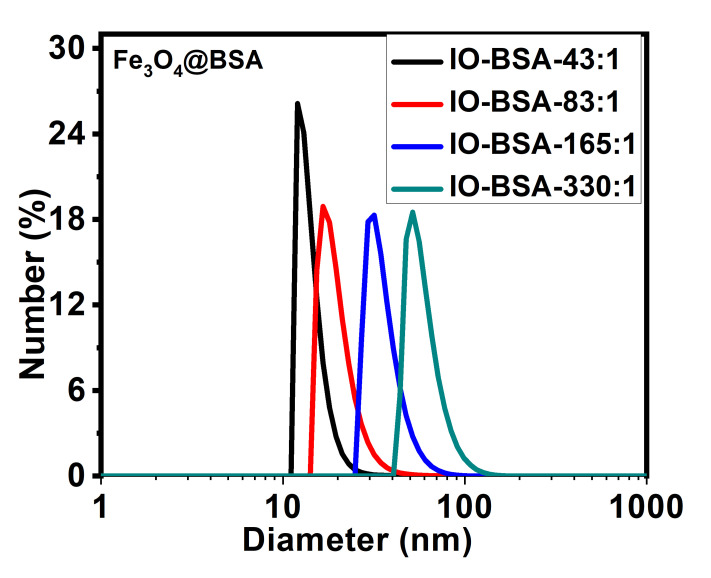
Hydrodynamic diameter of Fe_3_O_4_@BSA nanoparticles with different Fe:BSA molar ratios.

The water dispersibility was further evaluated by dynamic light scattering (DLS). As shown in Figure 9, a single peak was observed for all nanoparticles and the hydrodynamic size was relatively small. For example, Fe_3_O_4_@BSA of different Fe:BSA molar ratios—43:1, 83:1, 165:1 and 330:1—has a hydrodynamic size of 14.4, 20.1, 36.1 and 60.0 nm respectively. The hydrodynamic size of the nanoparticles in deionized water reduces with increasing BSA in the Fe:BSA molar ratio (43:1 < 83:1 < 165:1 < 330:1), which agrees with the TEM data. The relatively small hydrodynamic sizes further support the excellent water dispersibility of the nanoparticles.

### 3.7. Relaxivity Studies

Iron oxide nanoparticles hold great potential as contrast agents for MRI. One of the advantages of iron oxide nanoparticles is biocompatibility, which has been proven by clinical usage. In MRI, contrast agents reduce the *T*_1_ and *T*_2_ relaxation times of nuclei in tissues. The *T*_1_ contrast agents have relative reduction in *T*_1_, while *T*_2_ contrast agents have relative reduction in *T*_2_. Their efficiency is measured in terms of their relaxivity values (*r*_1_ and *r*_2_) [43]. The longitudinal relaxation time (*T*_1_) and transverse relaxation time (*T*_2_) of Fe_3_O_4_@BSA and Fe_2_O_3_@BSA were evaluated using an MRI scanner at 3T. When the graph of 1/*T*_1_ and 1/*T*_2_ was plotted against iron concentration, the slopes *r*_1_ and *r*_2_ were obtained respectively.

**Figure 10:**
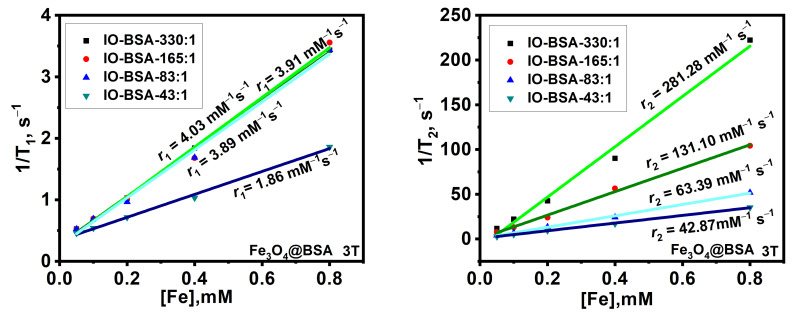
The plot of 1/*T*_1_, s^−1^ and 1/*T*_2_, s^−1^ against [Fe], mM of Fe_3_O_4_@BSA at different Fe-to-BSA molar ratios using a 3T relaxometer.

The size of nanoparticles has a great impact on their relaxivity and MRI performance. The representative plots are shown in Figure 10. For Fe_3_O_4_@BSA with Fe:BSA molar ratios of 42.5:1, 82.5:1, 165:1 and 330:1, the *r*_1_ values are 1.86, 3.89, 4.03 and 3.91 mM^−1^ s^−1^ respectively, and the *r*_2_ values are 42.87, 63.39, 131.10 and 281.28 mM^−1^ s^−1^ respectively.

For Fe_3_O_4_@BSA, the *r*_2_/*r*_1_ ratios at Fe:BSA molar ratios of 330:1, 165:1, 83:1, and 43:1 are listed in Table 1. The *r*_2_/*r*_1_ ratio generally decreases with decreasing particle size, and the smallest ratio was observed at 83:1. Interestingly, the ratio increased again to 43:1 despite this sample having the smallest size (3.7 ± 0.8 nm). This may result from the large amount of BSA coating at this ratio, which can limit interactions between the magnetic core and surrounding water molecules [44].

The *r*_2_/*r*_1_ ratios of Fe_2_O_3_@BSA were also investigated and are shown in Table 2. As expected, the *r*_2_/*r*_1_ ratios of Fe_2_O_3_@BSA were consistently lower than those of Fe_3_O_4_@BSA. Similar to Fe_3_O_4_@BSA, the smallest *r*_2_/*r*_1_ ratio (11.52) was observed at 83:1. The *r*_2_/*r*_1_ value of 11.52 is relatively low and is almost the same as the previously reported value (10.6) for BSA-coated iron oxide nanoparticles [31]. The *r*_2_/*r*_1_ ratio for Ferumoxyltol was reported to be 12.3, which is higher than the value obtained in this study [45]. The small *r*_2_/*r*_1_ ratio of Fe_2_O_3_@BSA synthesized in this work demostrate the potential for *T*_1_-weighed MRI.

### 3.8. Phantom Study

**Figure 11:**
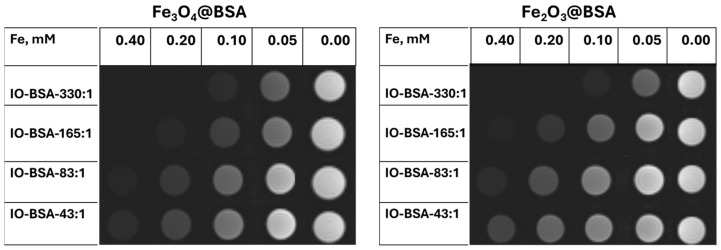
*T*_2_ phantom images of Fe_3_O_4_@BSA and Fe_3_O_4_@BSA with different Fe:BSA ratios using a 3T scanner.

We further conducted phantom studies of the iron oxide nanoparticles in solution. As shown in Figure 11, *T*_2_ phantom images were acquired for Fe_3_O_4_@BSA and Fe_2_O_3_@BSA with different Fe:BSA ratios. With increases in concentrations, the imaging became dark. The imaging was darker at the same concentration for larger nanoparticles. The results are consistent with the high *r*_2_. Although the nanoparticles have small sizes, those with Fe:BSA molar ratios of 165:1 and 330:1 exhibited noticeable *T*_2_ effects at higher concentrations. Consistent with other iron oxide nanoparticles, the BSA-coated nanoparticles could potentially be used as *T*_2_ contrast agents [14].

**Figure 12:**
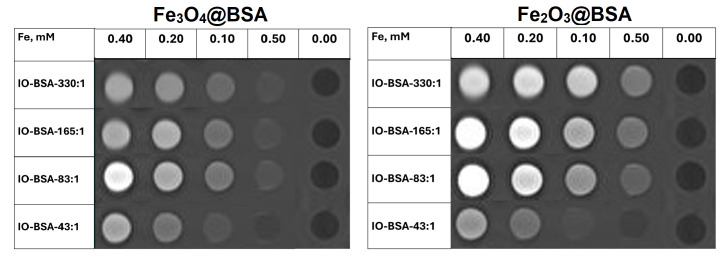
*T*_1_ phantom images of Fe_3_O_4_@BSA and Fe_2_O_3_@BSA with different Fe:BSA molar ratios using a 3T scanner.

Due to the small sizes, our BSA-coated iron oxide nanoparticles can be used as high-performance *T*_1_ contrast agents. The *T*_1_ phantom images are shown in Figure 12. All nanoparticles at different concentrations displayed a *T*_1_ brightening effect. For Fe_3_O_4_@BSA, the 83:1 Fe:BSA ratio exhibited the strongest *T*_1_ brightening effect and the lowest *r*_2_/*r*_1_ ratio. For Fe_2_O_3_@BSA, the 83:1 Fe:BSA ratios showed enhanced *T*_1_ brightening and correspondingly lower *r*_2_/*r*_1_ ratios compared to the other samples. These phantom studies clearly demonstrate that the BSA-coated nanoparticles are stable and suitable for *T*_1_-weighted MRI applications. A *T*_1_-weighted contrast agent is usually favorable for clinical use.

## 4. Conclusions

In this study, we demonstrated that the size of iron oxide nanoparticles can be effectively controlled using a simple co-precipitation method by adjusting the Fe:BSA molar ratio. Increasing the amount of BSA led to smaller nanoparticles. XRD analysis confirmed that the nanoparticles are highly crystalline, with peak intensities increasing with particle size. FT-IR and UV-Vis spectroscopy verified successful BSA coating on the nanoparticle surfaces. The resulting Fe_2_O_3_@BSA nanoparticles exhibited excellent water dispersibility across different pH and high-ionic-strength solutions.

Oxidation of Fe_3_O_4_@BSA to Fe_2_O_3_@BSA enhanced the *T*_1_ contrast properties of the nanoparticles, with *r*_2_/*r*_1_ ratios lower than those of Fe_3_O_4_@BSA. Phantom studies further confirmed the stability and *T*_1_ imaging capability of the BSA-coated nanoparticles. The value is comparable to the lowest *r*_2_/*r*_1_ ratios reported in the literature.

Compared to conventional thermal decomposition methods, which require organic solvents and high temperatures, this co-precipitation approach uses water as the solvent and mild reaction conditions. The method is low-cost, non-toxic, and time-efficient, making it a practical and scalable strategy for producing ultra-small, biocompatible iron oxide nanoparticles suitable for biomedical imaging applications.

## Figures and Tables

**Table 1:** The *r*_2_/*r*_1_ ratio of Fe_3_O_4_@BSA with different Fe:BSA ratios at 3T field strength.

Fe:BSA Molar Ratio	*r*_1_, mM^−1^ s^−1^	*r*_2_, mM^−1^ s^−1^	*r*_2_/*r*_1_
330:1	3.91	281.28	71.93
165:1	4.03	131.10	32.53
83:1	3.86	63.39	16.42
43:1	1.86	42.87	23.05

**Table 2:** The *r*_2_/*r*_1_ ratio of Fe_2_O_3_@BSA with different Fe:BSA ratios at 3T field strength.

Fe:BSA Molar Ratio	*r*_1_, mM^−1^ s^−1^	*r*_2_, mM^−1^ s^−1^	*r*_2_/*r*_1_
330:1	9.18	279.29	30.42
165:1	5.79	76.58	13.23
83:1	3.94	45.38	11.52
43:1	1.24	26.61	21.45

## Data Availability

The original contributions presented in this study are included in the article. Further inquiries can be directed to the corresponding author.
